# Associations between sociodemographic exposures, growth and development during infancy with development at the age of eight years among children: Analysis of a maternal education trial in rural Uganda

**DOI:** 10.7189/jogh.14.04228

**Published:** 2024-12-06

**Authors:** Paul Kakwangire, Moses Ngari, Grace Muhoozi, Ane Cecilie Westerberg, Prudence Atukunda, Per Ole Iversen

**Affiliations:** 1 Department of Nutrition, IMB, University of Oslo, Oslo, Norway; 2 Department of Family Life and Consumer Studies (Home Economics), Kyambogo University, Kampala, Uganda; 3 KEMRI Wellcome Trust Research Programme, Kilifi, Kenya and Department of Public Health, School of Health and Human Sciences, Kilifi, Kenya; 4 Division of Obstetrics and Gynecology, Department of Obstetrics, Oslo University Hospital, Rikshospitalet, Oslo, Norway; 5 School of Health Sciences, Kristiania University College, Oslo, Norway; 6 Center for Crisis Psychology, Faculty of Psychology, University of Bergen, Bergen, Norway; 7 Department of Haematology, Oslo University Hospital, Oslo, Norway; 8 Division of Human Nutrition, Stellenbosch University, Tygerberg, South Africa

## Abstract

**Background:**

Links between early life exposures and child development later in life are not sufficiently explored in low- and middle-income countries. We studied associations between sociodemographic variables, growth and development at six to eight months with developmental outcomes at eight years.

**Methods:**

We used data from a maternal education trial which included 511 mother-infant pairs at children’s age of six to eight months (baseline). In this follow-up study, data from 361 mother-child pairs were available. Questionnaires were used to collect sociodemographic variables. Growth (anthropometry) was measured by study personnel and converted to z-scores according to the World Health Organization (WHO) growth reference. Child development (cognitive, motor and language) at baseline was assessed using Bayley Scales of Infant and Todler Development, third edition (BSID-III). Development at eight years was measured using two neuropsychological tools: Kaufman Assessment Battery for Children Second Edition (KABC-II) and Test of Variables of Attention (TOVA).

**Results:**

Higher weight-for-age z-scores (adjusted odds ratio (aOR) = 0.74; 95% confidence interval (CI) = 0.53, 0.98; *P* = 0.04), better maternal education (aOR = 0.86; 95% CI = 0.78, 0.96; *P* = 0.03). and better household head education (aOR = 0.86; 95% CI = 0.78, 0.96; *P* = 0.03) at six to eight months of age were associated with lower odds of scoring below average on KABC-II categorical scores at eight years of age. Motor composite scores and maternal parity at six to eight months of age were positively associated with auditory and visual TOVA scores (all *P*-values <0.05) at eight years. Cognitive composite scores at six to eight months of age were positively associated with visual TOVA scores (*P* < 0.05). In contrast, weight-for-length z-scores and household head age were negatively associated with both auditory and visual TOVA scores (*P* < 0.05). Being a female child was associated with lower auditory and visual TOVA scores (*P* < 0.05).

**Conclusions:**

At six to eight months of age, growth and development, gender, maternal education and parity, and household head age and education were associated with child development at eight years. Interventions emphasising improved growth and development in infancy, as well as parental educational attainment, may improve long-term developmental outcomes.

The United Nations Sustainable Development Goal 4 emphasises the role of early childhood development as vital, not only for the health and well-being of individuals, but also progress at national and global levels [1]. Despite this, inadequate childhood development persists in low- and middle-income countries (LMICs), with millions of children at high risk of not attaining their full developmental potential [2]. Child development is influenced by a complex interaction between nature and nurture, including physical, social and biological factors [3,4]. Poverty and adverse experiences in early childhood, combined with undernutrition and poor health, may influence developmental trajectories and mental health, and thus affect the overall health and well-being of an individual throughout the life-course [2,5,6]. As a LMIC, Uganda faces high rates of undernutrition with almost one in every three children being stunted (low height/length-for-age z-score and a proxy for chronic undernutrition) [7]. Earlier evidence indicated inadequate child development promotion in Uganda reporting a big proportion of children unable to receive stimulation like learning activities and toys [8].

Brain growth and development occur in the first years of life, making this period crucial in the life of an individual [9,10]. Childhood development is a process of maturation that results from ordered advancement of cognitive, socioemotional, motor, and language skills, which are built on the foundational abilities laid in early life [11]. Indeed, any developmental delays in early life can negatively impact educational attainments, human capital, and occupational outcomes later in life [12,13]. Furthermore, development issues in early life may hamper emotional well-being and behaviour later [14]. Notably, early identification and treatment of any developmental issues can prevent short- and long-term health effects [15,16].

Comprehensive assessment of childhood developmental aspects requires various age-appropriate psychometric tools. These tools, however, are not widely available in LMICs, and even when they are, they tend to be costly and are mainly used in research settings [17]. Therefore, it is important to explore alternative ways of predicting childhood development through identifying possible developmental determinants. These determinants may help highlight targets for interventions to promote development. Much of the data available on determinants of childhood development are obtained in high-income countries [18], and only limited information is available in LMICs.

Between 2013 and 2014, we conducted the Child Nutrition and Development (CHNUDEV) study, a two-armed cluster-randomised controlled trial (cRCT) in rural Southwestern Uganda [19]. The main aims were to examine the effects of a six months’ nutrition, hygiene and stimulation education intervention among mothers of six to eight months old children, on child growth-, developmental- and maternal mental health outcomes; with assessments at two [20], three [21], six [22] and eight years of age [23]. At the trial baseline, we assessed child, maternal, and household characteristics. Here we assessed the associations between these baseline characteristics and childhood development at eight years of age.

## METHODS

### Participants and study area

The CHNUDEV trial recruited mothers and their infants aged six to eight months between October 2013 and February 2014 in the rural districts of greater Kabale (sub-counties of Butanda, Ikumba, Kamuganguzi, Kamwezi, Muko, and Ruhija) and Kisoro (sub-counties of Muramba, Nyakabande, Nyarubuye, and Nyarusiiza) in Southwestern Uganda **(**Figure S1 in the **Online Supplementary Document**). This area is largely occupied by peasant farmers who survive on subsistence agriculture. We excluded children with congenital malformations or physical handicap that would influence food intake as well as those with mental- or brain illnesses.

### Sampling and sample size

Details on the sampling and sample size calculation have been described elsewhere [20] and in the **Online Supplementary Document**. Briefly, we performed simple random sampling to allocate 10 sub-counties (clusters) in both districts (six from Kabale and four from Kisoro) to either the intervention or control group. The original trial had at least 80% power to detect a difference of 0.3 standard deviations (SDs) in height-for-age z-score between the intervention and control group. In this secondary analysis including 361 children, assuming alpha of 0.05 and intra-cluster correlation of 0.01, the analysis had >80% power to detect protective effect of higher education (at least secondary education vs. none/primary education) by at least 50% (i.e. adjusted odds ratio (aOR) of 0.5) on the binary KABC-II outcome (lower odds of being below average to lower extreme KABC-II score). We used computer generated random numbers to randomly select the participating villages from sub-counties. We then used complete enumeration to select households from which the youngest eligible child was recruited. For twins, we randomly selected one of them.

### Intervention activities in the original cRCT

The cRCT intervention is detailed elsewhere [20] and in the Methods section of the **Online Supplementary Document**. Bachelor graduate nutritionist and a psychologist delivered nutrition, hygiene, oral health, and child stimulation education content to groups of mothers, each with a village health team member as a group leader. This education package was delivered using behavioural change communication strategies and demonstrations with emphasis on the appropriate complementary feeding, nutritious food preparation, proper hygiene practices (including oral health), and child play and stimulation. The education sessions were delivered monthly for six months followed by monthly booster sessions until children were three years of age. The leader followed up the group members and encouraged them to adhere to the intervention.

### Baseline data collection

A semi-structured questionnaire was used to collect sociodemographic data including maternal, household and child characteristics. Trained research assistants administered questionnaires to the mothers or main caregivers through interviews. Child growth was assessed using anthropometric measurements, including weight, length, head circumference, and mid-upper arm circumference, taken using calibrated tools following World Health Organization (WHO) recommendations [24]. The measurements were then combined into anthropometric indices (weight-for-length z-scores, weight-for-age z-scores, and length-for-age z-scores) based on WHO growth standards [24].

A poverty score card for Uganda was administered to assess the socioeconomic status of the participants [25]. The poverty scores were then summed and arranged by poverty likelihood on a scale of 0 to 100, with a score of 70.8 or higher considered extreme poverty. Child diet was assessed based on a dietary diversity score tool which consists of eight food groups and has been validated for use in similar settings [26]. The food groups include grains; roots or tubers; vitamin A-rich plant foods; other fruits or vegetables; meat, poultry, fish, seafood; eggs; pulses/legumes/nuts; milk and milk products; and foods cooked in oil/fat. Each of the food groups consumed by a child was given a score of one. Child cognitive, motor, and language development were assessed using Bayley Scales of Infant and Toddler Development, third edition (BSID-III) [27].

### Assessment of outcomes at eight years

The main outcome in the current follow-up study was childhood development assessed using two separate neuropsychological tools: the Kaufman Assessment Battery for Children Second Edition (KABC-II) and Test of Variables of Attention (TOVA).

#### KABC-II

KABC-II is a test of cognitive and processing capabilities administered to individuals aged 3–18 years [28]. This test has been previously validated for use in a similar Ugandan setting [29]. We used the Cattle-Horn-Carroll (CHC) model of KABC-II to assess the children [28]. The model is based five scales including: short-term memory, visual processing, long-term storage and retrieval, fluid reasoning, and crystallised ability. We summarised the scores from these five scales into a single global scale called the Fluid Crystallized Index (FCI) (Table S1 in the **Online Supplementary Document**). We then grouped the FCI scores into five categories: upper extreme (score ≥131), above average (116–130), average (85–115), below average (70–84), and lower extreme (≤69) [28].

#### TOVA

The TOVA test is a computerised visual and auditory continuous performance test that measures attention and impulse control in five areas: response time variability (a measure of how consistent the child’s speed is in responding correctly), mean response time (a measure of the average amount of time a child takes to correctly respond from when correct stimuli are presented), commission errors (the total number of incorrect responses to the incorrect stimuli divided by the number of non-target stimuli presented), omission errors (total incorrect non-responses to the target stimulus divided by the sum of target stimuli presented), and d-prime (highlights the child’s accuracy in differentiating target and non-target stimuli) (**Online Supplementary Document**). Visual and auditory tests were conducted for all the children and each of the tests lasted 22 minutes. The TOVA device, version 9.1 (TOVA Company LLC, San Diego, CA, USA) records the performance of each child and generates a number of response parameters. One of these performance parameters are z-scores which we considered for this study [30]. The z-scores are computed by comparing individual child performances with a normative sample (children of the same age and sex without attention deficits) and presented on all the five TOVA areas [30,31]. Higher z-score represents better performance across all five domains. The TOVA test has previously been used in settings similar to our study area and has proved to be reliable in assessing attention and attention deficits [32,33].

Details about KABC-II and TOVA and how the assessments were conducted were recently published elsewhere [23].

### Statistical analyses

We assessed statistical significance using a two-tailed *P*-value <0.05 and cluster-adjusted 95% confidence intervals (CI). To correct for the multiple correlated outcomes, all the regression analysis *P*-values were adjusted for false discovery rate (FDR) using the Benjamini-Hochberg procedure [34]. Because ~ 1/3 of the originally recruited children had missing outcomes data that could not be assumed to be missing at random, we did not include them in current analyses. We present characteristics for both the original cohort (n = 511) and the current cohort (n = 361). The KABC-II scores and the visual TOVA omission error scores were log transformed because they were not normally distributed.

We assessed the association between all baseline characteristics collected and childhood development (KABC-II and TOVA continuous scores) at eight years using multilevel linear regression models with the sub-county as random intercept (to account for the clustering). We used linear regression models because the outcomes were continuous variables standardised or log-transformed to obtain normalised values. We also assessed these associations using categorical KABC-II scores. For the categorised KABC-II scores, we collapsed the five FCI categories into a binary variable (average to upper extreme vs. below average to lower extreme). We then conducted mixed effect multilevel logistic regression (because the outcome was binary), adjusting for clustering at sub-county level to assess the association between baseline characteristics and KABC-II scores ‘below average to lower extreme’. In both the linear and logistic regression models, we used multilevel/random effects approach to explicitly correct for the correlations within each cluster since the original study was a cRCT.

All baseline exposures collected in the original trial were included as independent variables in all the regression models. To build multivariable regression models (both linear and logistic), we retained independent variables with a *P*-value <0.1 using backward stepwise approach. However, in both bivariable and multivariable models, we adjusted for the randomisation study group (i.e. intervention or control) as *a priori* confounder to account for the effect of the intervention on the psychometric scores [23]. For all the linear regression models, we checked for multicollinearity among all the exposures using variance inflation factor (VIF) and its inverse (1/VIF, the tolerance). Weight-for-age z-scores were found to have high collinearity with both height-for-age and weight-for-length z-scores and was excluded in all linear multivariable models based on a rule of thumb of VIF value >10 and tolerance <0.01 (Tables S5–6 in the **Online Supplementary Document**). To test if the residuals had a mean of zero and a normal distribution after running the multivariable linear regression models, we visually inspected the kernel density and performed the Shapiro-Wilk test for normality on all the models’ residuals (Figure S2 in the **Online Supplementary Document**). The final multivariable logistic regression was internally validated using bootstrapped area under receiver operating characteristic curve with 1000 resampling with replacement. We also internally validated all the multivariable linear regression models by visually inspecting both the models’ residuals variance and their 1000 resampling with replacement bootstrapped residuals variance (Table S7 in the **Online Supplementary Document**).

We performed statistical analyses using Stata, version 17.0 (StataCorp LLC, College Station, TX, USA).

## RESULTS

### Inclusion and study group characteristics

**Figure 1:**
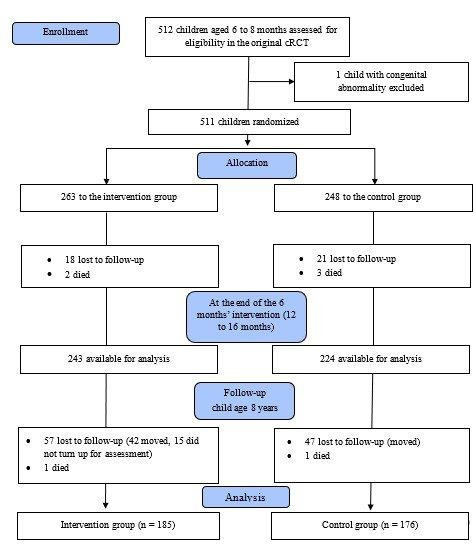
Flowchart of the inclusion process.

We enrolled 511 children aged six to eight months in the original trial, of whom 263 were allocated to the intervention group and 248 to the control group. In the current follow-up study, we included data from 361 (71%) children aged eight years (intervention: n/N = 185/361, 51%; control: n/N = 176/361, 49%) in our analyses (**Figure 1**).

Apart from child dietary diversity scores in the follow-up cohort, there were no other significant differences in the child, maternal, or household characteristics between the intervention and the control groups in the original trial or the follow-up cohort (**Table 1**). Child growth and development characteristics among the intervention and control groups were similar for both study cohorts. No harm was detected in any group.

**Table 1:** Baseline characteristics of the study participants*

	Original trial cohort (n = 511)		Follow-up study cohort (n = 361)		
	**Intervention (n = 263)**	**Control (n = 248)**	**Intervention (n = 185)**	**Control (n = 176)**	***P*-value**†
**Child characteristics**					
Child sex					
*Male*	139 (53)	123 (50)	95 (51)	89 (51)	0.90‖
*Female*	124 (47)	125 (50)	90 (49)	87 (49)	
Child age in months, x̄ (SD)	7.4 (0.8)	7.3 (0.9)	7.4 (0.9)	7.3 (0.9)	0.28¶
Exclusive breastfeeding					
*Yes*	184 (70)	178 (72)	124 (67)	130 (74)	0.42‖
*No*	79 (30)	70 (28)	61 (33)	46 (26)	
Length-for-age z-score, x̄ (SD)‡	−1.07 (1.15)	−1.21 (1.24)	−1.06 (1.20)	−1.17 (1.30)	0.39¶
Weight-for-age z-score, x̄ (SD)‡	−0.63 (1.10)	−0.72 (1.13)	−0.64 (1.10)	−0.70 (1.10)	0.64¶
Weight-for-length z-score, x̄ (SD)‡	0.12 (1.21)	0.15 (1.26)	0.10 (1.20)	0.16 (1.30)	0.76¶
Cognitive composite score, MD (IQR)§	100 (15)	105 (20)	100 (15)	105 (20)	0.43**
Language composite score, MD (IQR)§	104 (14)	100 (14)	103 (21)	100 (20)	0.14**
Motor composite score, MD (IQR)§	105 (14)	104 (15)	105 (21)	103 (21)	0.61**
Child dietary diversity score, x̄ (SD)	3.3 (1.6)	2.8 (1.6)	3.3 (1.7)	2.7 (1.6)	<0.001¶
**Maternal characteristics**					
Maternal age in years, MD (IQR)	25 (9)	26 (9)	26 (8)	27 (8)	0.37**
Maternal education level					
*None/primary*	173 (66)	166 (67)	123 (67)	125 (71)	0.83‖
*Lower secondary*	64 (24)	62 (25)	47 (25)	39 (22)	
*Tertiary*	26 (10)	20 (8)	15 (8)	12 (7)	
Maternal parity					
*<5 children*	187 (71)	184 (74)	129 (70)	119 (68)	0.66‖
*≥5 children*	76 (29)	64 (26)	56 (30)	57 (32)	
Household characteristics					
Household head age in years, MD (IQR)	30 (11)	30 (13)	30 (10)	30 (13)	0.17**
Household number of years in school, x̄ (SD)	6.2	3.1	6.1	3.0	0.11¶
Poverty score, MD (IQR)	49 (17)	49 (18)	49 (13)	49 (17)	0.55**
Household member size					
*3–5 members*	150 (57)	139 (56)	105 (57)	92 (52)	0.39‖
*6–13 members*	113 (43)	109 (44)	80 (43)	84 (48)	

IQR – interquartile range, MD – median, SD – standard deviation, x̄ – mean

*Presented as n (%) unless specified otherwise.

†The *P*-values (cluster-adjusted) compare the two study groups in the follow-up cohort.

‡The anthropometrical z-scores were calculated based on the WHO growth standards.

§The developmental composite scores were assessed based on Bayley Scales of Infant and Toddler Development Test-III.

‖χ^2^ tests.

¶T-tests.

**Rank sum tests.

### Associations between baseline characteristics at six to eight months of age and binary KABC-II at eight years of age

After adjusting for confounders, baseline factors – including weight-for-age z-scores, maternal education and household head education – were significantly associated with lower odds of KABC-II scores ‘below average and lower extreme’ (**Table 2**). Every unit increase in weight for-age z-score at baseline was associated with a 26% decrease in the odds of being classified as below average and lower extreme KABC-II category (aOR = 0.74; 95% CI = 0.53, 0.98; *P* = 0.04). Relative to children whose mothers had no education or only primary education, those whose mothers had secondary education had 14% lower odds of being categorised as below average and lower extreme on KABC-II (aOR = 0.86; 95% CI = 0.78, 0.96; *P* = 0.03). Similarly, the education of the household head had a significant association with the child KABC-II performance, every extra year of schooling reduced the odds of the child being in the below average and lower extreme KABC-II category by 14% (aOR = 0.86; 95% CI = 0.78, 0.96; *P* = 0.03). Exclusive breastfeeding, BSID-composite scores (cognitive and language) and poverty scores had significant associations with KABC-II at bivariable, but not at the multivariable level of analysis (Table S2 in the **Online Supplementary Document**).

**Table 2:** Associations between baseline characteristics and the KABC-II scores ‘below average and lower extreme’*

	Bivariable analysis			Multivariable analysis		
**Variable**	**Crude OR**	**95% CI**	***P*-value**	**aOR**	**95% CI**	***P*-value**
Study group						
*Control*	1.0	ref		1.0	ref	
*Intervention*	0.08	0.03, 0.20	<0.001	0.10	0.04, 0.28	<0.001
Child sex					-	
*Male*	1.0	ref			-	
*Female*	0.92	0.50, 1.70	0.78		-	
Age in months	1.15	0.79, 1.66	0.47		-	
Exclusive breastfeeding						
*No*	1.0	ref		1.0	ref	
*Yes*	2.15	1.12, 4.13	0.02	1.86	0.91, 3.80	0.09
Height-for-age z-scores	0.84	0.65, 1.09	0.19		-	
Weight-for-age z-scores	0.67	0.50, 0.89	0.007	0.74	0.53, 0.98	0.04
Weight-for-length z-scores	0.78	0.60, 1.01	0.06		-	
Cognitive composite score	0.97	0.95, 0.99	0.02		-	
Language composite score	0.98	0.95, 0.99	0.03	0.98	0.95, 1.01	0.06
Motor composite score	0.99	0.97, 1.02	0.65		-	
Maternal age in years	1.01	0.96, 1.07	0.65		-	
Maternal education						
*None/primary level*	1.0	ref		1.0	ref	
*Lower secondary*	0.49	0.25, 0.94	0.03	0.47	0.21, 0.97	0.03
*Tertiary*	0.39	0.14, 1.09	0.07	0.52	0.15, 1.79	0.30
Maternal parity						
*Less than five children*	1.0	ref			-	
*Five or more children*	0.99	0.51, 1.94	0.99		-	
Household head age in years	1.01	0.96, 1.04	0.97		-	
Household head number of years in school	0.86	0.77, 0.95	0.005	0.86	0.78, 0.96	0.03
Household size						
*3 to 5*	1.0	ref			-	
*6 to 13*	0.94	0.51, 1.74	0.85		-	
Poverty score	0.97	0.94, 0.99	0.04		-	
Child dietary diversity score	0.85	0.71, 1.02	0.08		-	
Multivariable model performance						
*Bootstrapped AUC (95% CI)*				0.82	0.76, 0.88	

aOR – adjusted odds ratio, AUC – area under receiver operating characteristic curve, CI – confidence interval, OR – odds ratio, ref – reference

*Odds ratios are from a mixed-effect multilevel regression model. The outcome variable was binary KABC-II scores categorised into average to upper extreme vs. below average to lower extreme.

### Association between baseline characteristics and auditory TOVA

Child age (time span between six and eight months) at baseline was significantly associated with mean response time in that for every extra month in child age added at baseline, the mean response time z-score increased by 0.16 units at eight years (coefficient = 0.16; 95% CI = 0.02, 0.30; *P* = 0.04). Child sex, weight-for-length z-scores and motor composite scores were significantly associated with commission error z-scores (a measure of impulsivity based on responses to the non-target) at eight years. Girls’ commission error z-score points were 0.18 lower than those of the boys at eight years (*P* < 0.001). For every unit increase in baseline weight-for-length z-score, commission error z-scores went down by 0.33 points (*P* < 0.001). Every unit increase in baseline motor composite score led to a 0.02 increase in commission error z-scores at eight years (*P* = 0.04) (**Table 3**).

**Table 3:** Multivariable analysis of baseline characteristics associated with auditory TOVA*

	Auditory TOVA mean response time z-scores		Auditory TOVA response time variability z-scores		Auditory TOVA commission errors z-scores (inhibition/impulsivity)		Auditory TOVA omission errors z-scores (sustained attention/inattention)		Auditory TOVA D-prime z-scores (ability to differentiate between target and non-target stimuli)	
**Variable**	**Adjusted *β *(95% CI)**	**FDR-adjusted *P*-value**	**Adjusted *β *(95% CI)**	**FDR-adjusted *P*-value**	**Adjusted *β *(95% CI)**	**FDR-adjusted *P*-value**	**Adjusted *β *(95% CI)**	**FDR-adjusted *P*-value**	**Adjusted *β *(95% CI)**	**FDR-adjusted *P*-value**
Study group										
*Control*	ref		ref		ref		ref		ref	
*Intervention*	0.40 (0.15, 0.66)	0.008	0.61 (0.27, 0.96)	<0.001	0.99 (0.25, 1.74)	0.02	1.28 (0.67, 1.89)	<0.001	0.54 (0.25, 0.83)	<0.001
Child sex										
*Male*	ref		ref		ref		ref		ref	
*Female*	−0.18 (−0.42, 0.07)	0.20	−0.17 (−0.38, 0.04)	0.23	−1.38 (−1.84, −0.92)	<0.001	−0.28 (−0.66, 0.11)	0.191	0.01 (−0.14, 0.16)	0.88
Age in months	0.16 (0.02, 0.30)	0.04	0.059 (−0.06, 0.18)	0.49	−0.04 (−0.31,023)	0.78	0.23 (0.01, 0.45)	0.04	0.05 (−0.03, 0.14)	0.33
Weight-for-length z-scores	**-**		-		−0.33 (−0.51, −0.14)	<0.001			−0.09 (−0.16, −0.04)	0.006
Motor Composite Score	**-**		-		0.02 (0.0001, 0.03)	0.04			0.22 (0.01, 0.43)	0.04
Maternal Education										
*None/primary level*	**-**		ref		-		-		-	
*Lower secondary*	**-**		0.07 (−0.20, 0.33)	0.49	-		-		-	
*Tertiary*	**-**		−0.33 (−0.75, 0.08)	0.24	-		-		-	
Maternal parity										
*Less than five children*	**-**		**-**		**-**		ref		**-**	
*Five or more children*	**-**		**-**		**-**		0.52 (0.04, 0.99)	0.04	**-**	
Household head age in years	**-**		**-**		**-**		−0.02 (−0.05, −0.001)	0.04	**-**	
Poverty score	−0.01 (−0.01, 0.01)	0.86	0.003 (−0.006, 0.01)	0.49	0.01 (−0.01, 0.03)	0.41	−0.002 (−0.019, 0.016)	0.87	0.003 (−0.003, 0.011)	0.33

*β* – correlation coefficient, CI – confidence interval, FDR – false discovery rate, ref – reference, TOVA – test of variables of attention

*Full list of exposure variables explored in the regression analysis are shown in Table S3 in the **Online Supplementary Document**. Here we only report independent variables selected for inclusion in multivariable models.

Child age, mother’s number of biological children, and mother’s household head age were significantly associated with omission error z-scores (a measure of inattention based on lack of response to the target stimuli) at eight years. An extra month added to a child at baseline was associated with a 0.23 increase in omission error z-score at eight years (*β*  = 0.23; 95% CI = 0.012, 0.45; *P* = 0.04). Children born to mothers of five or more children at baseline performed better than those of mothers having less than five children (β = 0.52; 95% CI = 0.044, 0.99; *P* = 0.04). The older the household head was at baseline, the lower the omission error z-score attained by the child at 8 years (β = −0.024; 95% CI = −0.047, −0.001; *P* = 0.04).

Weight-for-length z-scores and motor composite scores were significantly associated with D-prime z-scores, the ratio between the rate of correct responses, and responses to non-target at eight years. Higher baseline weight-for-length z-scores were associated with reduced D-prime z-scores (*P* = 0.006) and higher baseline motor composition scores were associated with higher D-prime z-scores at eight years (*P* = 0.044). We did not find any baseline factors associated with response time variability z-scores at eight years.

### Association between baseline characteristics and visual TOVA

Baseline motor composite scores and mother’s number of biological children had significant associations with mean response time z-scores at eight years (**Table 4**). For every point increase in baseline motor composite scores, mean response time z-scores increased by 0.02 points (*P* = 0.04). Children born to mothers of five children or more at baseline had significantly higher mean response time z-scores at eight years of age (β = 0.59; 95% CI = 0.16, 1.01; *P* = 0.02). For every unit increase in the baseline cognitive composite score, the response time variability z-score increased by 0.02 (*P* = 0.008). Similarly, cognitive composite score and child sex had significant associations with commission error z-scores at 8 years. Higher baseline cognitive composite scores were associated with higher commission error z-scores (β = 0.02; 95% CI = 0.002, 0.03; *P* = 0.04). Being a girl at baseline was associated with lower commission (β = −0.83; 95% CI = −1.21, −0.45; *P* < 0.001) and omission error z-scores (β = −0.35; 95% CI = −0.43, −0.28; *P* < 0.001) at eight years of age. D-prime z-scores at eight years were significantly associated with baseline child sex, weight-for-length z-scores and cognitive composite scores. Being a female (β = −0.56; 95% CI = −0.70, −0.43; *P* = 0.02) and higher weight-for-length z-scores (β = −0.11; 95% CI = −0.18, −0.04; *P* = 0.04) at baseline were associated with reduced D-prime z-scores. For every baseline cognitive composite score added, the child D-prime z-score increased by 0.01 points (*P* = 0.02).

**Table 4:** Multivariable analysis of baseline characteristics associated with visual TOVA z-scores*

	Visual TOVA mean response time z-scores		Visual TOVA response time variability z-scores		Visual TOVA commission errors z-scores (inhibition/impulsivity)		Log visual TOVA omission errors z-scores (sustained attention/inattention)		Visual TOVA D-prime z-scores (ability to differentiate between target and non-target stimuli)	
**Variable**	**Adjusted *β *(95% CI)**	**FDR-adjusted *P*-value**	**Adjusted *β *(95% CI)**	**FDR-adjusted *P*-value**	**Adjusted *β *(95% CI)**	**FDR-adjusted *P*-value**	**Adjusted *β *(95% CI)**	**FDR-adjusted *P*-value**	**Adjusted *β *(95% CI)**	**FDR-adjusted *P*-value**
Study group										
*Control*	ref		ref		ref		ref		ref	
*Intervention*	0.73 (0.21, 1.25)	0.02	1.01 (0.44, 1.58)	<0.001	0.86 (0.27, 1.45)	0.001	0.13 (0.05, 0.21)	0.003	0.45 (0.25, 0.66)	<0.001
Child sex										
*Male*	ref		ref		ref		ref		ref	
*Female*	−0.17 (−0.53,0.19)	0.35	−0.07 (−0.47, 0.34)	0.75	−0.83 (−1.21, −0.45)	<0.001	−0.35 (−0.43, −0.28)	<0.001	−0.56 (−0.70, −0.43)	0.02
Age in months	0.16 (−0.052, 0.37)	0.21	0.109 (−0.126, 0.346)	0.61	0.06 (−0.16, 0.29)	0.57	0.03 (−0.01, 0.079)	0.16	0.05 (−0.03, 0.13)	0.21
Weight-for-length z-scores	-								−0.11 (−0.18, −0.04)	0.04
Cognitive composite score	-		0.02 (0.01, 0.04)	0.008	0.02 (0.002, 0.03)	0.04			0.01 (0.001, 0.012)	0.02
Motor composite score	0.016 (0.002, 0.029)	0.04								
Maternal education										
*None/primary level*	-				ref					
*Lower secondary*	-				−0.17 (−0.64, 0.31)	0.57				
*Tertiary*	-				0.66 (−0.08, 1.40)	0.14				
Maternal parity										
*Less than five children*	ref									
*Five or more children*	0.59 (0.16, 1.01)	0.02								
Poverty score	**-**		−0.01 (−0.02, 0.01)	0.74	−0.01 (−0.03, 0.01)	0.33	0.0002 (−0.003, 0.004)	0.92	0.001 (−0.004, 0.007)	0.58
Child dietary diversity score	0.01 (−0.01, 0.03)	0.21							−0.03 (−0.08, 0.01)	0.17

*β* – correlation coefficient, CI – confidence interval, FDR – false discovery rate, ref – reference, TOVA – test of variables of attention

*Full list of exposure variables explored in the regression analysis are shown in Table S4 in the **Online Supplementary Document**. Here we only report independent variables selected for inclusion in multivariable models

## DISCUSSION

We assessed associations between baseline characteristics of the CHNUDEV child participants at six to eight months of age and their development at eight years of age using KABC-II and TOVA. After adjustments, baseline factors including weight-for-age z-scores, maternal education, and household head education were significantly associated with binary KABC-II scores. Child age (time span of six to eight months), child sex, weight-for-length z-scores, motor composite scores, maternal parity and household head age were significantly associated with auditory TOVA scores. In addition, baseline motor composite scores, cognitive composite scores, maternal parity, child sex and weight-for-length z-scores were significantly associated with visual TOVA scores.

Children who had better nutritional status (using weight-for-age z-scores as proxy) and higher developmental scores (cognitive and motor) at baseline, performed better at eight years than their counterparts with worse nutritional status and lower developmental scores. It is well known that an early childhood environment that promotes good health and nutrition coupled with learning, stimulation, protection and emotional support, can play a key role in later life development [16]. Conversely, adverse early life exposures may exert long-term effects mediated at least partly via epigenetic pathways, causing differences in phenotypes of individuals that may persist across the lifespan [35]. This has been termed as ‘programming’, where environmental factors impact on body structure and function causing differences in phenotypes independent of the genotype [35–37]. Moreover, the positive association between nutritional status (i.e. weight-for-age z-scores) and binary KABC-II scores is in agreement with a number of previous findings [38]. For example, severe malnutrition among children, especially severe stunting, and severe underweight can impair child cognitive, motor and behavioural outcomes [5,38–40].

Interestingly, weight-for-length z-scores were negatively associated with both visual and auditory TOVA scores on three distinct scales (auditory commission errors, auditory D-prime and visual D-prime). Higher weight-for-length z-scores may reflect increased body fat percentage which has previously been negatively associated with child development [38], possibly linked to less physical activity or poor nutrition quality. Reduced physical activity may negatively impact child development [41]. Low weight-for-length, an indicator for acute malnutrition, is also a criterion for admitting children into therapeutic feeding centers in Uganda. In these centers, however, one of the treatment components is child stimulation; it is therefore possible that children with lower weight for height z-scores, if admitted, could have benefited from extra stimulation that they received during their treatment [42]. Previous studies in similar settings have reported no effect of weight- for-length z-scores on various child developmental domains [43,44].

Being a girl was consistently associated with low scores on various TOVA tests. This could be attributed to the fact that boys and girls are brought up differently. In our study area, parents tend to depict boys as active and hence encourage them to play more than they do to girls. Girls are taught to be humble and disciplined which sometimes discourages them from engaging in play activities [45]. These gender role stereotypes have been known to affect child development for many years [46]. Gender norms and beliefs tend to favor boys in many aspects [47]. According to one cognitive development theory, a child as an agent of their own gender role socialisation [48], highlighting that gender roles motivate children to participate in gender-specific activities. Consequently, once children fully identify themselves with a particular gender, they positively value activities that are consistent with that gender identity [48]. Our results are, however, in disagreement with some previous studies that have reported girls to have a slight advantage in terms of development [49,50].

Maternal education and parity were associated with child development. Mothers are usually the main caregivers and therefore play a significant role in the growth and development of their children. The positive association of maternal education and child development is consistent with previous findings [51]. The more years the mother attends school, the more likely it is that she interacts with and stimulates the child and hence better child development among more educated mothers [51]. We found a positive association between maternal parity (number of biological children born to the mother) and TOVA scores. The more children the mother has, the bigger the household size. More children living together are likely to interact and play together, which may improve development. The number of household members was positively correlated with better child mental health at various stages in life elsewhere [52]. In Bangladesh, however, maternal parity was found to negatively impact early childhood development [53].

We also found household head factors like age and education associated with child development. Fathers are in most cases the heads of the households in our study area. Fathers play and interaction with children at an early age is reported to promote child developmental aspects of emotion, social and cognition [54].

Our study has several strengths. The child development assessors were blinded to the study group allocation, thus minimising measurement bias. Child development was assessed using two independent and comprehensive neuropsychological tools (KABC-II and TOVA) that have been used and validated in settings similar to our study area. Data collection both at baseline and at eight years was done by trained personnel including graduate psychologists and nutritionists. Lastly, most previous child development studies have focused on a single aspect of child development. In contrast, our study comprehensively reports on child developmental predictors based on a number of parameters, including processing and cognitive skills (KABC-II) and attention and inhibitory control (TOVA). Our study also has some limitations. Nearly 30% of the children from the original cRCT were not available for the current follow-up study and this could have reduced our power to assess some associations. No data imputation was done on the records’ missing outcomes because we did not assume that these outcomes were missing at random. In addition, the follow-up study was observational and examines only associations, hence we were not able to study causality. Furthermore, we did not have data on mother-child interactions which could play a role in child development. We used questionnaires for baseline assessment and hence we cannot rule out the possibility of recall bias in some aspects like child dietary diversity assessment.

## CONCLUSIONS

At six to eight months of age, infant growth and development, gender, maternal education, maternal parity, household head age and education, were associated with child development outcomes at eight years. Interventions emphasising improved growth in infancy, parental educational attainment and gender equality may improve long-term developmental outcomes. Our study shows direction for future studies aiming to investigate early determinants of child development, as well as interventions aiming to improve child development in low resource settings such as Uganda. These results can be used by government ministries and organisations working on childhood development. More research i warranted to further understand these associations and identify causal relationships, while similar studies in the future should include assessment of the interaction between mothers/caretakers and their children.

## Additional material


Online Supplementary Document
