# Supplementary material for: Associations between sociodemographic exposures, growth and development during infancy with development at the age of eight years among children: Analysis of a maternal education trial in rural Uganda
Source: J Glob Health. 2024 Dec 6;14:04228. doi: 10.7189/jogh.14.04228 (PMC11622353; doi:10.7189/jogh.14.04228)
Supplement: Online Supplementary Document [file jogh-14-04228-s001.pdf]

Supplementary Document

Kakwangire et al.:

Associations between sociodemographic exposures, growth and development during infancy with development at 8 years: Analysis of a maternal education trial in rural Uganda

Table of Contents

Supplementary Methods 2
The original cluster-randomized controlled trial (cRCT) 2
Sample size calculation in the original cRCT 2
The education intervention in the original cRCT 3
Booster sessions of the educational components after the original intervention period 5
Child development assessment tools at 8 years 5
The Kaufman Assessment Battery for Children, Second Edition (KABC-II) 5
Test of Variables of Attention (TOVA) 7
Supplementary Results 9
Supplementary References 20

Supplementary Methods

The original cluster-randomized controlled trial (cRCT)

Figure S1. Visual map describing the CHNUDEV study area.

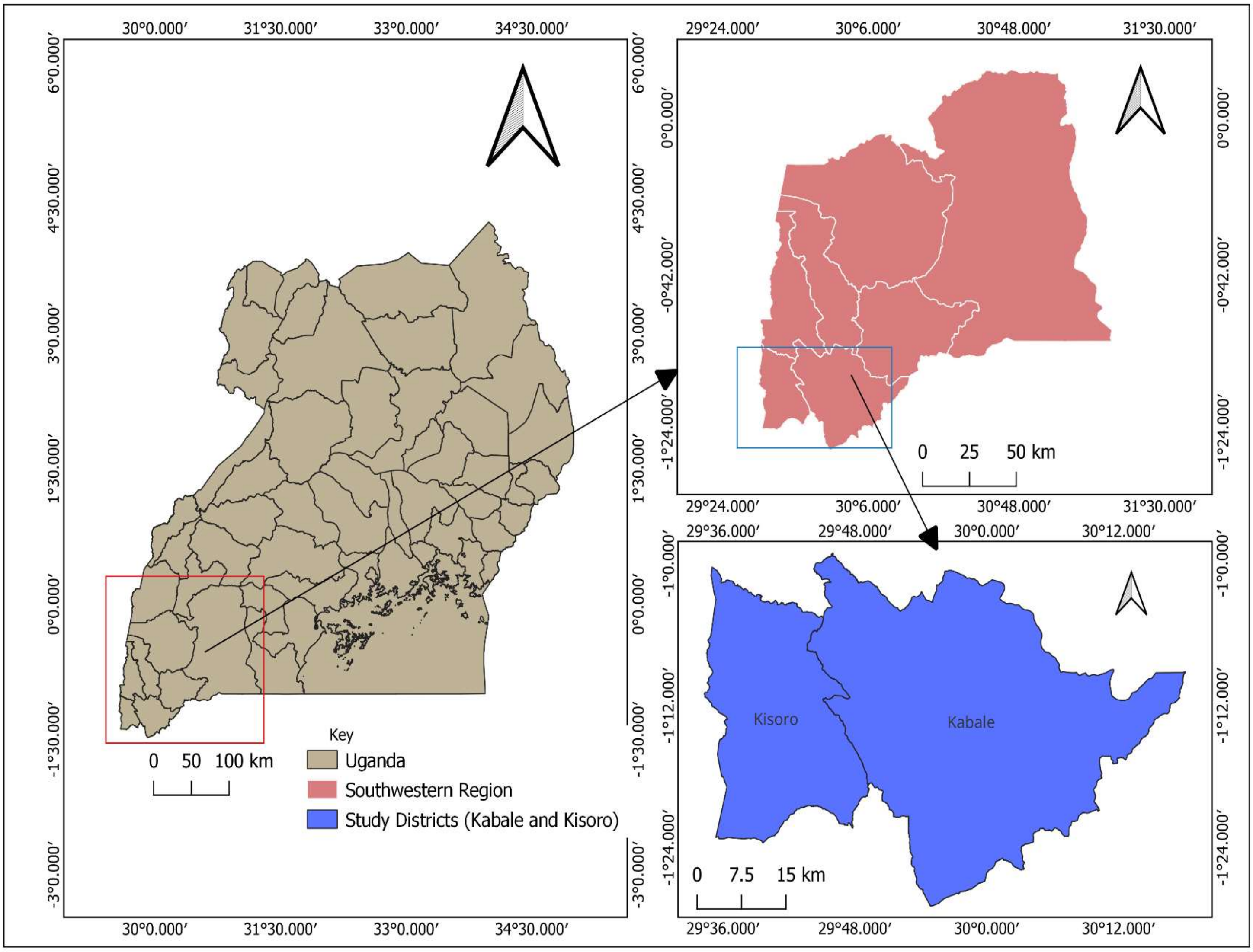

Sample size calculation in the original cRCT

In the original trial, sample size was calculated based on the primary outcome, height-for-age z-score (HAZ) at child age of 20–24 months. The mean ± SD for HAZ is 0.0 ± 1.0 in a healthy population. We defined a HAZ difference of 0.3 SD between the two study groups as clinically relevant. To detect the 0.3 SD difference in HAZ at 5% significance level and 80% power, we

needed 176 children in each study arm. We therefore recruited fifty-one children per sub-county presuming 10 sub-counties as clusters and an intra-cluster correlation of 0.01. The analyses were by intention-to-treat.

The education intervention in the original cRCT

The intervention mainly focused on nutrition, hygiene and stimulation education and it was delivered to mothers/caretakers. Also, demonstrations to mothers were done on food preparation, good hygiene practices and making of play materials using locally available items. The intervention was delivered through regular meetings for six months by a nutrition education team. Participating mothers were organised in groups and each group was led by a member of the village health team. The main role of the group leader was to keep reminding the mothers of the key intervention aspects with an aim of promoting behaviour change. The aspects of the intervention are described below.

Nutrition education

This package mainly focused on the PAHO/WHO guiding principles of complementary feeding (1). Our intervention put emphasis on the following:

- The advantages of continued breastfeeding.
- The proper way of positioning and attaching the baby on the breast.
- The composition of both fore and hind breast milk and the importance of each to the baby.
- Breastfeeding cues and the frequency of breastfeeding, including night feeds.
- Timely complementary feeding as breastfeeding alone is no longer sufficient at six months of age to supply all the nutritional requirements.
- The importance of texture and consistence of food at different stages.
- The advantages of feeding a child on a variety of foods from different food groups.
- The correct feeding frequency at different stages.
- Active feeding and its importance especially for sick children.

Cooking demonstrations

- Preparation and cooking of complementary dishes locally called 'ekitoobero'.
- Preparation of enriched porridge using local cereals, animal source food and vegetable oil.
- Use of the small silver fish to enrich various complementary food foods with animal protein.

- Using soy milk and soy powder in the preparation of complementary foods.
- Use of eggs as a key source of protein.

Hygiene education

The following were emphasized;

- Keeping homes and home environments clean at all times.
- Hand washing after visiting a pit latrine, before eating, and after any other activities that leave hands contaminated.
- Always preparing food in a clean and hygienic environment.
- To always avoid feeding the child leftover food that is improperly kept and not reheated.
- Good oral hygiene for every household member; we provided toothbrushes to all members of the participating households and we showed them how to correctly brush their teeth.

Child stimulation

We emphasized the following;
What household members can do to help children play. We taught them the key child developmental domains including cognitive-, motor- and communication development.

The role of play in developing child creativity and social skills was emphasized (2). We encouraged the mothers and other family members to teach their children how to identify body parts through educating and asking the children to name different parts (3). We taught them the hide and seek strategies of child stimulation; we taught them to hide the child favourite items and ask the child to find the items. We further demonstrated to the mothers how to make toys locally, how to engage in imaginary plays, plus the importance of screwing and unscrewing techniques in child stimulation.

We taught them the importance of both verbal and nonverbal communication aspect of language development (4). The "We Talk" slogan was used to emphasize that talking to the child regularly helps them to learn as they attempt to talk back (4). We encouraged them to make use of imitation, roleplaying, music and singing for communication skills development (5).

For motor development, we emphasised the "Learn whereas playing" slogan. We explained the concept of gross motor as the use of body control and coordination to enhance accuracy, speed, and security skills (6). We further highlighted fine motor skills as the ability to perform more

complicated tasks like tying shoelaces, writing, and unbuttoning clothes (5).

Booster sessions of the educational components after the original intervention period

After the 6 months' intervention, we conducted booster sessions three times every month to the intervention groups until when the children made three years of age with an aim of improving adherence to the intervention activities. During booster sessions, mothers/caretakers were reminded about key messages of the intervention relating to nutrition, hygiene, food preparation and child stimulation. These sessions were conducted by the CHNUDEV education team and each session lasted about 6 hours.

Child development assessment tools at 8 years

The Kaufman Assessment Battery for Children, Second Edition (KABC-II)

Our assessments were based on the Cattle-Horn-Carroll model that uses the Fluid-Crystallized index (FCI) as we recognize the importance of crystallized knowledge in cognitive functioning. In addition, evidence suggests that FCI is a better predictor of achievement across various ages (7). Table S1 presents the scales under FCI and the subtests that we administered under each scale.

Table S1. FCI scales and subtests.

FCI scales	Subtests
• *Short-Term Memory*	• Word order<br>• Number Recall
• *Visual Processing*	• Block Counting<br>• Rover<br>• Triangles
• *Long-Term Storage and Retrieval*	• Atlantis<br>• Rebus subtests
• *Fluid Reasoning*	• Pattern Reasoning<br>• Story Completion.
• *Crystalized Ability*	• From Riddles<br>• Verbal Knowledge

Short-Term Memory subtests

- *Number Recall*: the examiner says a series of numbers and the child is then asked to repeat the numbers in the same order.
- *Word Order*: the child is presented with pictures of common objects. The examiner then mentions a series of the displayed objects and the child is asked to point at them in the same order.

Visual Processing subtests

- *Block Counting*: Pictures of various blocks stacked arranged in different ways are showed to the child and the child is asked to count the blocks in the pictures.
- *Rover*: A toy-dog (Rover) and a booklet with a grid on different pages are showed to the child. Each grid has a picture of a bone located at different points. The child is then asked to move the Rover from a specified starting point using the shortest route to the bone. The child also has to avoid a number of obstacles in the grid on the way to the bone.
- *Triangles*: The child being assessed is presented with various pictures of different shapes and a number of plastic and rubber shapes. The child is then asked to put together the provided shapes to match the pictures.

Long-Term Storage and Retrieval subtests

- *Atlantis*: Pictures of fish, plants and shells are presented to the child. Each picture is then given an imaginary name. The child is asked to identify various pictures from a number of them on a page.
- *Rebus*: The child is presented with a number of drawings and the examiner gives each drawing a name and asks the child to memorize the names. The examiner then touches a series of these drawings and asks the child to read them loud in phrase or sentence form.

Fluid Reasoning subsets

- *Pattern Reasoning*: A number of stimuli are presented to the child in a pattern form with some missing aspects. The child is asked to pick the missing stimulus from the provided options and complete the pattern.

- *Story completion*: A story book with a series of pictures on various pages is showed to the child being tested. The pictures are arranged in a story with some parts missing. The child is then given a set of cards with different pictures and asked to use the cards to fill the missing gaps in the story.

Crystalized Ability subsets

- Riddles: The examiner explains a key aspect of an object or an idea and the child is asked to name it.
- Verbal Knowledge: the child is showed a number of pictures and is asked to point to one that corresponds to a certain vocabulary word or one that responds to a certain question.

Test of Variables of Attention (TOVA)

Visual task

The visuals are of two big squares, one with a small dark square at the top and the other with a small dark square at the bottom. The child uses the dominant hand to hold a micro-switch and attentively observes the squares flashing on the screen. The child is told to press in the micro-switch as quickly as possible when the small dark square appears near the top. The child is also told to not respond when the small dark square appears near the bottom.

Auditory task

The auditory stimuli consist of two distinct tones (high and low notes) that are played through a speaker for the as the child attentively listens. The child is told to press the micro-switch as quickly as possible in response to high note and not to respond when a low note is played

Details of the TOVA variables

- *Response Time Variability:* This measures the variability in the child's response time for all the responses that are correct. It assesses how consistent the child is in responding to the target stimuli. The standard deviation of the mean correct responses is used to derive the response time variability scores.
- *Mean Response Time:* This quantifies the amount of time it takes for a child to make a correct response from when stimulus is presented. It assesses on average how long it takes a child to make correct responses to target stimuli. The mean response time score is obtained by adding

up all the correct response time and then dividing it by the number of correct presses on the micro-switch.

- *Commission Errors (COM):* The child is not expected to press the micro-switch button when an incorrect stimulus is presented. Commission error happens when the child fails to avoid responding to such an incorrect stimulus. The score is then calculated by adding up all the incorrect responses and dividing the total by the number of non-target stimuli presented.
- *Omission Errors (OE):* The child is expected to respond when a correct stimulus is presented. Omission error occurs when the child fails respond to a target stimulus. The score is computed from the total number non-responses divided by the total number of target stimuli presented.
- *D-prime:* This score is based on the Signal Detection Theory and it reflects the ratio between correct response rate and "false alarm rate". D prime is vital in identifying attention disorders. The score is computed from the child's ability to differentiate between target and non-target stimuli (8).

The details on how TOVA variables are mathematically calculated can be found in Leark et al. (8).

Supplementary Results

Table S2. Bivariable and multivariable analyses of the associations between baseline characteristics and continuous KABC II scores.

	**Bivariable analysis**			**Multivariable analysis**		
**Variable**	**Coefficient**	**95% CI**	***P*-value**	**Coefficient**	**95% CI**	**P-value**
**Nutrition education**						
Intervention	0.189	0.138, 0.239	0.000	0.176	0.121, 0.231	<0.001
**Child sex**						
Female	-0.009	-0.036, 0.018	0.513	-0.004	-0.031, 0.022	0.742
**Age in months**	0.002	-0.012, 0.018	0.720	0.001	-0.017, 0.014	0.871
**Exclusive Breastfeeding**						
Did exclusive breastfeeding	-0.031	-0.060, -0.001	0.041	-0.026	-0.055, 0.003	0.088
**Height-or-age z-scores**	0.009	-0.001, 0.020	0.090			
**Weigh-for-age z-scores**	0.013	0.001, 0.025	0.041			
**Weight-for-length z-scores**	0.003	-0.007, 0.014	0.528			
**Cognitive Composite Score**	0.001	0.001, 0.003	0.003			
**Language Composite Score**	0.001	0.0002, 0.002	0.013	0.001	0.0001, 0.002	**0.036**
**Motor Composite Score**	0.001	-0.001, 0.001	0.297			
**Maternal Age in years**	-0.001	-0.003, 0.001	0.386			
**Maternal Education**						
Lower secondary	0.041	0.009, 0.073	0.029	0.089	-0.004, 0.067	0.063
Tertiary	0.089	0.039, 0.140	<0.001	0.054	0.009, 0.120	**0.023**
**Mother's number of biological children**						
>= 5	-0.021	-0.049, 0.007	0.141			
**Household head number of years in school**	0.010	0.006, 0.015	0.000	0.006	0.001, 0.011	**0.019**
**Household size**						
6 to 13	-0.0148	-0.041, 0.012	0.278			
**Poverty score**	0.003	0.001, 0.004	0.000	0.002	0.001, 0.003	**0.003**
**Child dietary diversity score**	0.009	0.006, 0.017	0.035			

The multivariable analysis coefficients are presented after adjustments for Bayley Scales of Infant and Toddler Development Test-III language composite scores, maternal education, household head education and poverty score at 6-8 months. *P*-values are cluster-adjusted from multi-level linear regression and controlling for the intervention effect.

Table S3. Bivariable analyses of the associations between baseline characteristics and auditory TOVA z-scores.

	**Mean Response Time z-scores**		**Response time Variability**		**Commission Errors (inhibition/ impulsivity)**		**Omission Errors (sustained attention/inattention)**		**D-prime (ability to differentiate between target and non-target stimuli)**	
**Variable**	**Coefficient (95% CI)**	***P*-value**	**Coefficient 95% CI**	***P*-value**	**Coefficient 95% CI**	***P*-value**	**Coefficient 95% CI**	***P*-value**	**Coefficient 95% CI**	***P*-value**
**Study group**										
Intervention	0.43 (0.18,0.67)	0.001	0.63 (0.29,0.96)	<0.001	1.01 (0.13,1.89)	0.02	1.36 (0.72,1.10)	<0.001	0.56 (0.28,0.84)	<0.001
**Child sex**										
Female	-0.18 (-0.43,0.06)	0.14	-0.19 (-0.40,0.02)	0.08	-1.46 (-1.91-1.01)	<0.001	-0.30 (-0.68,0.09)	0.13	0.01 (-0.14,0.16)	0.90
**Age in months**	0.16 (0.02,0.30)	**0.03**	0.060 (-0.061,0.180)	0.33	0.03 (-0.24,0.30)	0.84	0.235 (0.016,0.455)	0.04	0.07 (-0.02,0.16)	0.12
**Exclusive breastfeeding**										
Did exclusive breastfeeding	-0.007 (-0.26,0.26)	0.96	0.08 (-0.16,0.31)	0.52	0.11 (-0.42,0.64)	0.68	0.05 (-0.38,0.48)	0.83	0.07 (-0.10,0.24)	0.41
**Height-for-age z-scores**	-0.04 (-0.14,0.06)	0.45	0.02 (-0.06,0.11)	0.62	0.07 (-0.12,0.27)	0.48	-0.02 (-0.18,0.14)	0.80	0.04 (-0.03,0.10)	0.27
**Weight-for-age z-scores**	-0.05 (-0.16,0.07)	0.43	-0.06 (-0.16,0.04)	0.26	-0.23 (-0.45-0.01)	**0.04**	-0.13 (-0.31,0.05)	0.15	-0.05 (-0.12,0.02)	0.17
**Weight-for-length z-scores**	-0.03 (-0.13,0.07)	0.52	-0.09 (-0.18-0.01)	**0.04**	-0.30 (-0.49-0.11)	**0.002**	-0.17 (-0.32-0.01)	**0.04**	-0.09 (-0.15-0.03)	**0.002**
**Cognitive composite score**	-0.001 (-0.010,0.009)	0.86	0.002 (-0.006,0.010)	0.60	0.017 (-0.001,0.036)	0.06	-0.01 (-0.02,0.01)	0.45	-0.001 (-0.006,0.005)	0.85
**Language composite score**	0.003 (-0.006,0.011)	0.53	-0.01 (-0.02-0.001)	0.03	0.003 (-0.013,0.020)	0.68	-0.01 (-0.02,0.003)	0.16	-0.002 (-0.007,0.002)	0.34
**Motor composite score**	-0.002 (-.010,0.007)	0.71	-0.00002 (-0.008,0.007)	0.99	0.018 (0.001,0.035)	**0.03**	-0.004 (-0.018,0.009)	0.54	0.00003 (-0.005,0.005)	0.99
**Maternal age in years**	-0.0001 (-0.020,0.020)	0.99	.0001 (-0.016,0.018)	0.94	-0.02 (-0.06,0.02)	0.41	-0.001 (-0.033,0.029)	0.92	0.002 (-0.010,0.014)	0.75
**Maternal education**										
Lower secondary	-0.01 (-0.30,0.29)	0.97	0.10 (-0.16,0.36)	0.44	0.33 (-0.25,0.91)	0.26	0.18 (-0.29,0.65)	0.46	0.10 (-0.09,0.28)	0.29
Tertiary	-0.24 (-0.71,0.23)	0.32	-0.29 (-0.70,0.11)	0.15	0.13 (-0.78,1.04)	0.78	-0.004 (-0.744,0.735)	0.99	-0.04 (-0.33,0.25)	0.77
**Mother's number of biological children**									-0.003 (-.011,0.004)	0.41
>= 5	0.12 (-0.14,0.38)	0.38	0.03 (-0.20,0.25)	0.81	0.07 (-0.44,0.57)	0.79	0.13 (-0.28,0.55)	0.53	0.11 (-0.05,0.27)	0.17
**Household head age in years**	-0.004 (-0.018,0.009)	0.55	-0.01 (-0.02,0.01)	0.32	-0.02 (-0.04,0.01)	0.23	-0.02 (-0.04,0.01)	0.14	-0.003 (-0.011,0.004)	0.41
**Household head number of years in school**	0.027 (-0.02,0.07)	0.21	0.02 (-0.02,0.05)	0.29	0.01 (-0.07,0.09)	0.70	0.05 (-0.01,0.12)	0.13	0.014 (-0.01,0.04)	0.28
**Household size**										
6 to 13	0.073 (-0.17,0.32)	0.56	0.06 (-0.15,0.27)	0.58	0.15 (-0.32,0.63)	0.53	-0.0003 (-0.386,0.385)	0.99	0.07 -0.08,0.23)	0.33
**Poverty score**	-0.0002 (-0.011,0.010)	0.97	0.002 (-0.007,0.012)	0.60	0.01 (-0.01,0.03)	0.40	0.001 (-0.016,0.018)	0.95	0.001 (-0.005,0.007)	0.75
			0.03 (-0.04,0.10)	0.39	0.03 (-0.12,0.18)	0.72	0.05 (-0.07,0.17)	0.41	0.03 (-0.02,0.08)	0.26

At bivariable analysis, weight-for-age z-scores, weight-for-length z-scores and Bayley Scales of Infant and Toddler Development Test-III motor composite scores were significantly associated with auditory TOVA scores.

Table S4. Bivariable analyses of the associations between baseline characteristics and visual TOVA z-scores

	**Mean Response Time Z-scores**		**Response time Variability**		**Commission Errors (inhibition/ impulsivity)**		**Omission Errors (sustained attention/inattention)**		**D-prime (ability to differentiate between target and non-target stimuli)**	
**Variable**	**Coefficient 95% CI**	***P*-value**	**Coefficient 95% CI**	***P*-value**	**Coefficient 95% CI**	***P*-value**	**Coefficient 95% CI**	***P*-value**	**Coefficient 95% CI**	***P*-value**
**Allocation arm**										
Intervention	0.76 (0.25,1.27)	0.004	0.94 (0.39,1.49)	0.001	0.80 (0.22,1.37)	0.01	0.14 (0.05,0.22)	0.002	0.46 (0.23,0.68)	<0.001
**Child sex**										
Female	-0.25 (-0.60,0.10)	0.17	-0.12 (-0.51,0.28)	0.56	-0.79 (-1.15,-0.43)	**<0.001**	-0.35 (-0.43, -0.28)	**<0.001**	-0.56 (-0.69,-0.42)	**<0.001**
**Age in months**	0.22 (0.02,0.42)	**0.031**	0.14 (-0.09,0.36)	0.23	0.05 (-0.16,0.26)	0.65	0.04 (-0.01,0.09)	0.10	0.08 (-0.004,0.16)	0.06
**Exclusive Breastfeeding**										
Yes	0.02 (-0.38,0 .41)	0.94	-0.15 (-0.58,0.28)	0.50	-0.36 (-0.77,0.05)	0.08	0.01 (-0.08,0.11)	0.75	-0.08 (-0.24,0.08)	0.34
**Height for Age Z-scores**	-0.03 (-0.17,0.12)	0.73	0.04 (-0.12,0.20)	0.59	0.10 (-0.06,0.25)	0.22	-0.001 (-0.036,0.034)	0.95	0.04 (-0.02,0.10)	0.21
**Weight for Age Z-scores**	-0.001 (-0.17,0.16)	0.99	-0.02 (-0.20,0.16)	0.86	-0.03 (-0.20,0.14)	0.71	-0.011 (-0.06,0.02)	0.42	-0.02 (-0.08,0.05)	0.64
**Weight for Length Z-scores**	-0.04 (-0.18,0.11)	0.63	-0.11 (-0.27,0.05)	0.17	-0.12 (-0.26,0.03)	0.12	-0.02 (-0.05,0.01)	0.24	-0.06 (-0.12,-0.002)	**0.04**
**Cognitive Composite Score**	0.016 (0.002,0.029)	**0.03**	0.02 (0.01,0.04)	**0.004**	0.02 (0.004,0.03)	**0.01**	0.002 (-0.001,0.005)	0.28	0.01 (0.002,0.01)	**0.005**
**Language Composite Score**	0.013 (0.001,0.025)	**0.04**	0.01 (-0.01,0.02)	0.26	0.001 (-0.011,0.014)	0.82	-0.0003 (-0.003,0.003)	0.86	0.001 (-0.004,0.005)	0.82
**Motor Composite Score**	0.010 (-0.002,0.023)	0.10	0.014 (0.001,0.028)	**0.03**	0.01 (-0.01,0.02)	0.38	0.002 (-0.001,0.005)	0.20	0.004 (-0.0001,0.010)	0.06
**Maternal Age**	0.02 (-0.01,0.05)	0.19	0.005 (-0.026,0.037)	0.73	-0.03 (-0.06,0.002)	0.07	0.001 (-0.006,0.008)	0.79	-0.002 (-0.014,0.009)	0.67
**Maternal Education**										
Lower secondary	0.16 (-0.27,0.58)	0.47	0.04 (-0.43,0.51)	0.87	-0.21 (-0.66,0.23)	0.35	0.04 (-0.07,0.14)	0.50	0.04 (-0.13,0.21)	0.66
Tertiary	0.26 (-0.42,0.93)	0.46	0.53 (-0.22,1.28)	0.17	0.53 (-0.18,1.23)	0.14	0.01 (-0.16,0.17)	0.94	0.13 (-0.14,0.40)	0.35
**Mother's number of biological children**										
>= 5	0.28 (-0.10,0.66)	0.15	0.16 (-0.26,0.58)	0.45	-0.18 (-0.58,0.22)	0.37	-0.003 (-0.095,0.089)	0.95	-0.04 (-0.194,0.11)	0.59
**Household head age**	0.007 (-0.01,0.03)	0.51	-0.003 (-0.025,0.018)	0.75	-0.01 (-0.03,0.01)	0.25	0.001 (-0.004,0.006)	0.59	-0.002 (-0.010,0.005)	0.59
**Household head number of years in school**	0.044 (-0.02,0.10)	0.16	0.02 (-0.05,0.09)	0.56	-0.03 (-0.09,0.04)	0.44	0.002 (-0.012, 0.016)	0.76	0.004 (-0.020,0.028)	0.73
**HH size**										
6 to 13	0.01 (-0.34,0.37)	0.94	0.17 (-0.22,0.56)	0.39	0.06 (-0.31,0.43)	0.77	-0.02 (-0.10,0.07)	0.72	0.003 (-0.14,0.15)	0.97
**Poverty score**	0.005 (-0.01,0 .02)	0.56	-0.0002 (-0.017,0.017)	0.98	-0.01 (-0.02,0.01)	0.54	0.001 (-0.003,0.004)	0.83	0.001 (-0.005,0.006)	0.83
**Child dietary diversity scores**	0.016 (-0.09,0.13)	0.77	0.01 (-0.11,0.13)	0.86	-0.04 (-0.16,0.06)	0.50	0.01 (-0.02,0.03)	0.66	0.0003 (-0.044,0.045)	0.98

At bivariable analysis, child sex, child age, Bayley Scales of Infant and Toddler Development Test-III cognitive composite scores, language composite scores, and motor composite scores were significantly associated with visual TOVA scores.

Table S5. Variance inflation factor (VIF) and Tolerance (1/VIF) of baseline characteristics assessed for associated with auditory TOVA.

	**Mean Response Time z-scores**		**Response time Variability**		**Commission Errors (inhibition/ impulsivity)**		**Omission Errors (sustained attention/inattention)**		**D-prime (ability to differentiate between target and non-target stimuli)**	
**Variable**	**VIF**	**1/VIF**	**VIF**	**1/VIF**	**VIF**	**1/VIF**	**VIF**	**1/VIF**	**VIF**	**1/VIF**
**Study arm**	5.38	0.19	5.34	0.19	5.31	0.19	5.31	0.19	5.31	0.19
**Child sex**	1.14	0.88	1.12	0.89	1.12	0.89	1.12	0.89	1.12	0.89
**Age in months**	1.16	**0.86**	1.18	0.85	1.17	0.85	1.17	0.85	1.17	0.85
**Exclusive breastfeeding**	1.12	0.89	1.12	0.89	1.12	0.89	1.12	0.89	1.12	0.89
**Height-for-age z-scores**	1.26	0.79	1.28	0.78	1.28	0.78	1.28	0.78	1.28	0.78
**Weight-for-age z-scores**	-		-		-		-		-	
**Weight-for-length z-scores**	1.23	0.81	1.22	**0.82**	1.22	**0.82**	1.22	**0.82**	1.22	**0.82**
**Cognitive composite score**	1.47	0.68	1.47	0.68	1.43	0.70	1.43	0.70	1.43	0.70
**Language composite score**	1.56	0.64	1.61	0.62	1.60	0.62	1.60	0.62	1.60	0.62
**Motor composite score**	1.64	0.61	1.63	0.61	1.63	**0.61**	1.63	0.61	1.63	0.61
**Maternal age in years**	2.89	0.35	3.00	0.33	3.0	0.33	3.0	0.33	3.0	0.33
**Maternal education**										
Lower secondary	1.29	0.77	1.35	0.74	1.35	0.74	1.35	0.74	1.35	0.74
Tertiary	1.28	0.78	1.30	0.77	1.30	0.77	1.30	0.77	1.30	0.77
**Mother's number of biological children**	2.20	0.46	2.17	0.46	2.17	0.46	2.17	0.46	2.17	0.46
**Household head age in years**	2.27	0.44	2.39	0.41	2.39	0.42	2.39	0.42	2.39	0.42
**Household head number of years in school**	1.33	0.75	1.53	0.65	1.52	0.66	1.52	0.66	1.52	0.66
**Household size**	2.33	0.43	2.29	0.44	2.29	0.44	2.29	0.44	2.29	0.44
**Poverty score**	1.79	0.56	1.88	0.53	1.88	0.53	1.88	0.53	1.33	0.75

VIF; variance inflation factor

Table S6. Variance inflation factor (VIF) and Tolerance (1/VIF) of baseline characteristics assessed for associated with visual TOVA z-scores.

**Variable**	**Visual TOVA Mean Response Time z-scores**		**Visual TOVA Response Time Variability z-scores**		**Visual TOVA Commission Errors z-scores (inhibition/ impulsivity)**		**Log Visual TOVA Omission Errors z-scores (sustained attention/inattent ion)**		**Visual TOVA D-prime z-scores (ability to differentiate between target and non-target stimuli)**	
	VIF	1/VI F	VIF	1/VIF	VIF	1/VIF	VIF	1/VIF	VIF	1/VIF
Study arm	5.29	0.19	5.29	0.19	5.29	0.19	5.33	0.19	5.33	0.19
Child sex	1.12	0.89	1.12	0.89	1.12	0.89	1.12	0.89	1.12	0.89
Age in months	1.17	0.85	1.17	0.85	1.22	0.82	1.18	0.85	1.18	0.85
Exclusive breastfeeding	1.11	0.90	1.11	0.90	1.11	0.90	1.11	0.90	1.11	0.90
Height-for-age z-scores	1.28	0.78	1.28	0.78	1.28	0.78	1.28	0.78	1.28	0.78
Weight-for-age z-scores	-		-		-		-		-	
Weight-for-length z-scores	1.22	0.82	1.22	0.82	1.17	0.85	1.22	0.82	1.22	0.82
Cognitive composite score	1.43	0.70	1.43	0.70	1.43	0.70	1.47	0.68	1.47	0.68
Language composite score	1.60	0.62	1.60	0.62	1.60	0.62	1.61	0.62	1.61	0.62
Motor composite score	1.63	0.61	1.63	0.61	1.63	0.61	1.63	0.61	1.63	0.61
Maternal age in years	3.01	0.33	3.01	0.33	3.01	0.33	3.01	0.33	3.01	0.33
Maternal education										
Lower secondary	1.35	0.74	1.35	0.74	1.35	0.74	1.35	0.73	1.35	0.73
Tertiary	1.30	0.77	1.30	0.77	1.30	0.77	1.30	0.77	1.30	0.77
Mother's number of biological children	2.17	0.46	2.17	0.46	2.17	0.46	2.17	0.46	2.17	0.46
Household head age in years	2.39	0.42	2.39	0.42	2.39	0.42	2.39	0.41	2.39	0.41
Household head number of years in school	1.52	0.66	1.52	0.66	1.52	0.66	1.53	0.65	1.53	0.65
Household size	2.28	0.44	2.28	0.44	2.28	0.44	2.28	0.43	2.28	0.43
Poverty score	1.87	0.53	1.87	0.53	1.87	0.53	1.87	0.54	1.87	0.54

VIF; variance inflation factor

Table S7. Internal validation of the multivariable linear regression models using Bootstrapping method.

Multivariable linear regression model	Models residuals variance	Bootstrapped residuals variance
**Auditory TOVA**		
**Auditory TOVA Mean Response Time z-scores**	1.37 (1.15–1.63)	1.37 (1.18–1.59)
**Auditory TOVA Response Time Variability z-scores**	1.01 (0.87–1.17)	1.01 (0.84–1.21)
**Auditory TOVA Commission Errors z-scores (inhibition)/impulsivity)**	0.58 (0.36–0.93)	0.58 (0.24–1.47
**Auditory TOVA Omission Errors z-scores (sustained attention/inattention)**	0.78 (0.52–1.16)	0.78 (0.42–1.44)
**Auditory TOVA D-prime z-scores (ability to differentiate between target and non-target stimuli)**	0.51 (0.44–0.59)	0.51 (0.41–0.62)
**Visual TOVA**		
**Visual TOVA Mean Response Time z-scores**	1.69 (1.31–2.14)	1.70 (1.29–2.17)
**Visual TOVA Response Time Variability z-scores**	1.42 (0.92–1.98)	1.41 (0.85–2.09)
**Visual TOVA Commission Errors z-scores (inhibition/ impulsivity)**	0.99 (0.57–1.49)	0.99 (0.25–1.98)
**Log Visual TOVA Omission Errors z-scores (sustained attention/inattention)**	0.08 (0.07–0.10)	0.08 (0.05–0.13)
**Visual TOVA D-prime z-scores (ability to differentiate between target and non-target stimuli)**	0.37 (0.31–0.42)	0.37 (0.31–0.43)

The results are residuals variance and 95% confidence intervals obtained from the final multivariable linear regression models.

Figure S2. Review of multivariable linear regression models residuals (Kernel density plots).

a. Auditory TOVA Mean Response Time z-scores Model

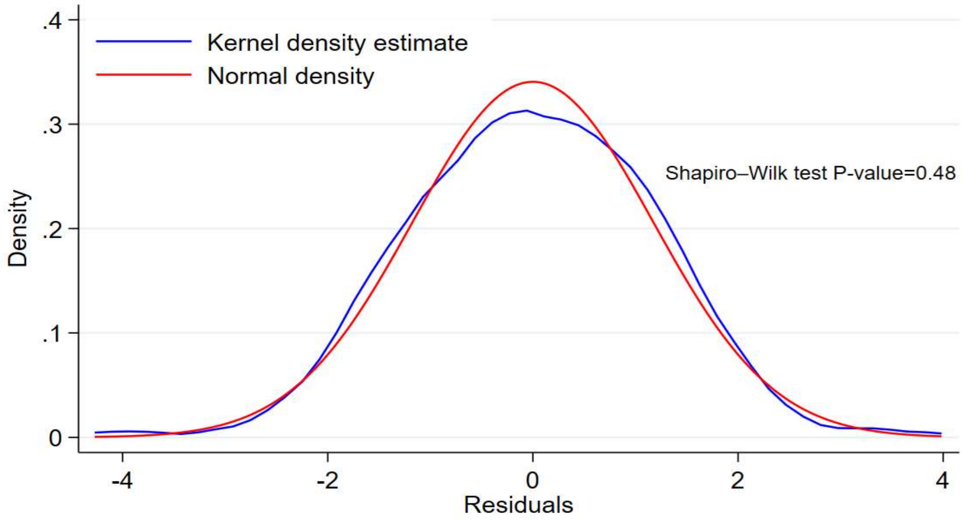

b. Auditory TOVA Response Time Variability z-scores Model

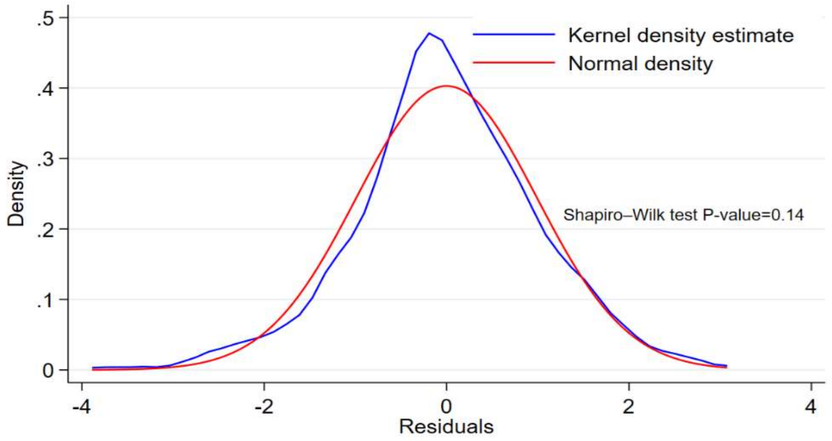

c. Auditory TOVA Commission Model z-scores Model

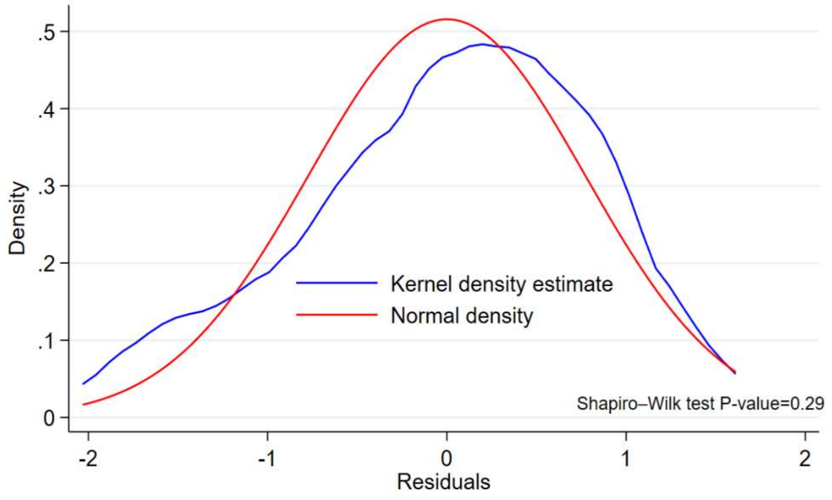

d. Auditory TOVA Omission Errors z- Model

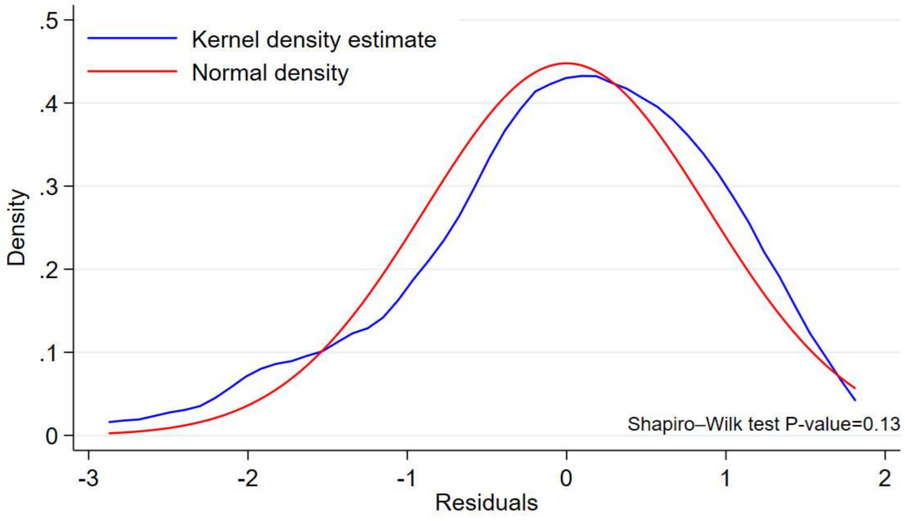

e. Auditory TOVA D-prime z-scores

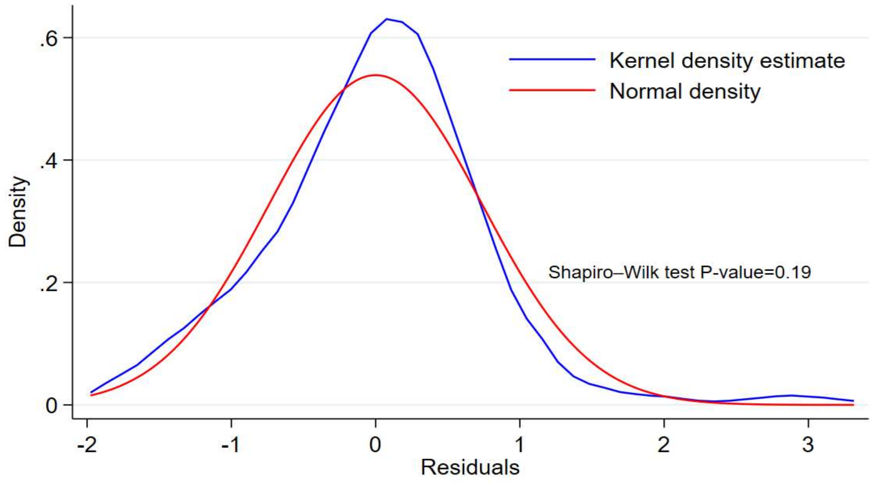

f. Visual TOVA Mean Response Time z-scores Model

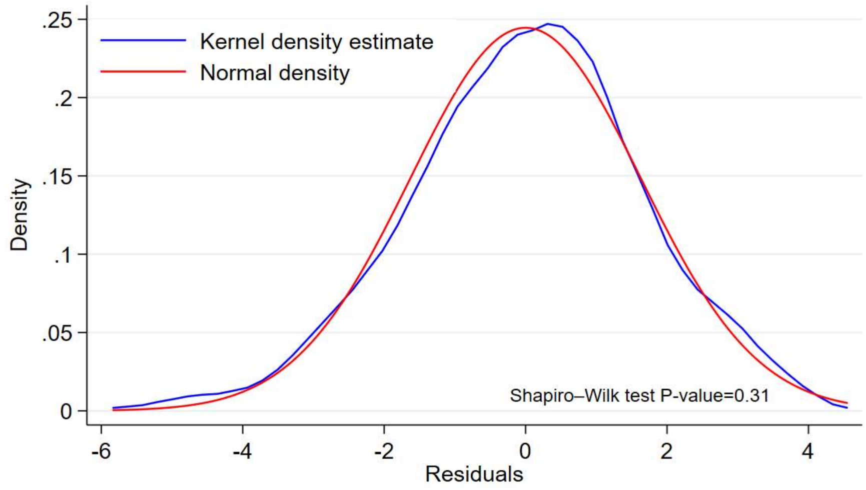

g. Visual TOVA Response Time Variability z-scores Model

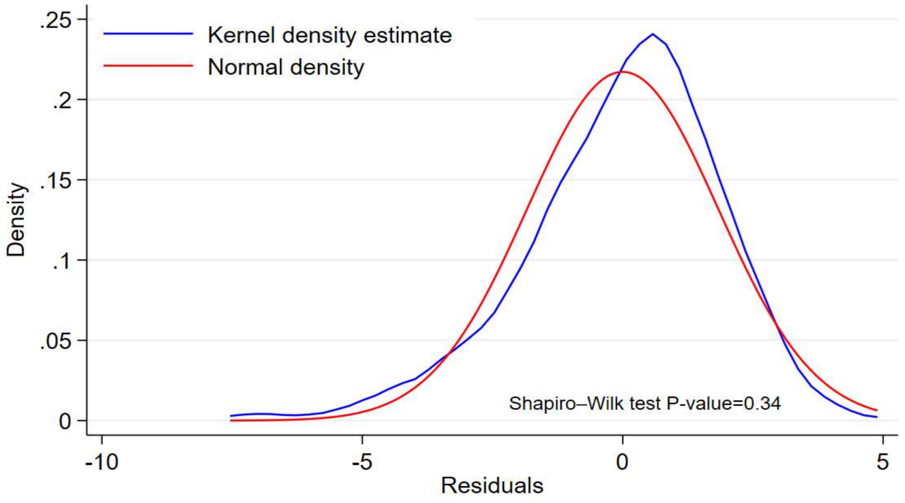

h. Visual TOVA Commission Errors z-scores Model

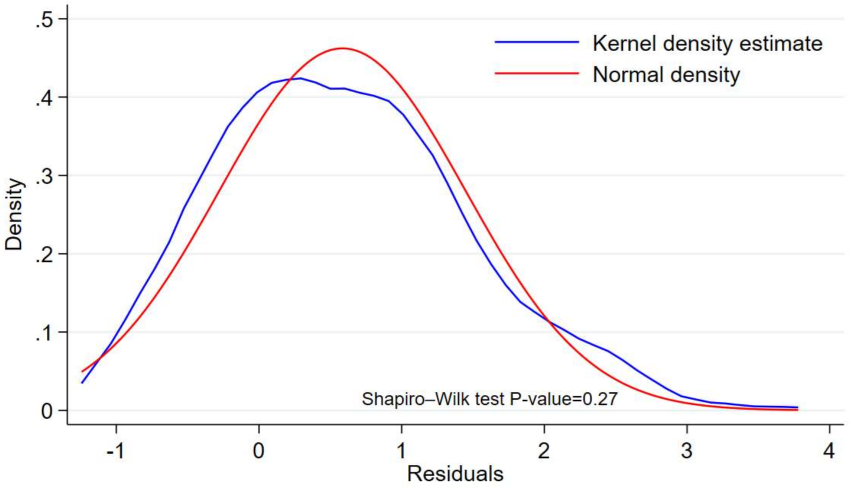

i. Log Visual TOVA Omission Errors z-scores Model

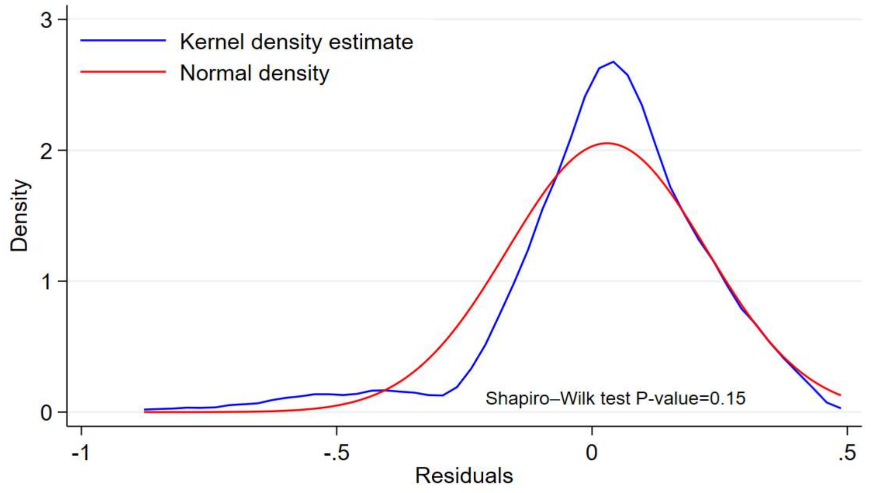

j. Visual TOVA D-prime z-scores Model

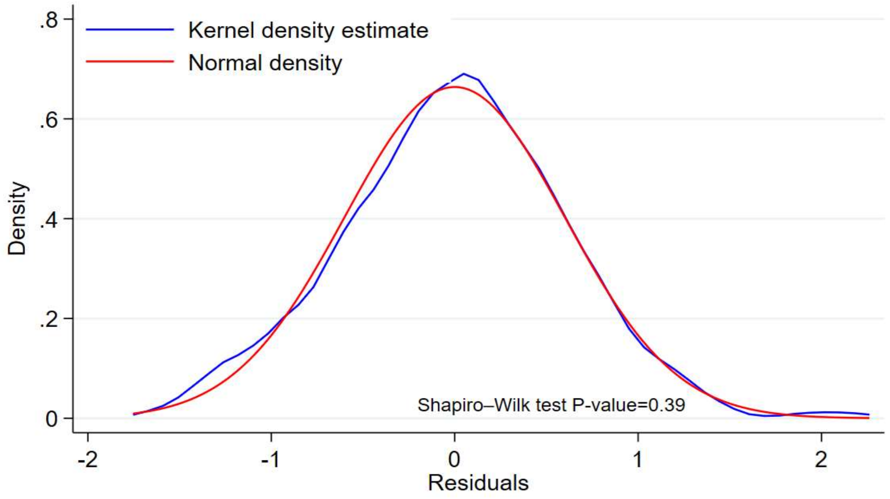

Supplementary References

1. Pan American Health Organization/World Health Organization. *Guiding principles for complementary feeding of the breastfed child.* Division of health promotion and protection. Washington/Geneva: PAHO, WHO, 2003.
2. Bruner, J.S. and Haste, H. *Making Sense (Routledge Revivals): The Child's Construction of the World*. 2010: Routledge.
3. Chang, S.M. et al., *Integrating a parenting intervention with routine primary health care: a cluster randomized trial.* Pediatrics, 2015. 136: 272-280.
4. Vos, R.C. et al., *Developmental trajectories of receptive and expressive communication in children and young adults with cerebral palsy.* Develop Med Child Neurol, 2014. 56: 951-959.
5. Hartinger, S.M. et al., *Impact of a child stimulation intervention on early child development in rural Peru: a cluster randomised trial using a reciprocal control design.* J Epidemiol Community Health, 2017. 71:217-224.
6. Donnelly, J.E. et al., *Physical activity, fitness, cognitive function, and academic achievement in children: a systematic review.* Med Sci Sports Exerc, 2016. 48: 1197.
7. Scheiber, C. and Kaufman, A.S. *Which of the three KABC-II global scores is the least biased?* J Ped Neuropsychol, 2015. 1: 21-35.
8. Leark, R.A. et al., *Test of Variables of Attention-TOVA Professional Manual.* 2020.
